# Comparative Analysis of Outer Membrane Vesicle Isolation Methods With an *Escherichia coli tolA* Mutant Reveals a Hypervesiculating Phenotype With Outer-Inner Membrane Vesicle Content

**DOI:** 10.3389/fmicb.2021.628801

**Published:** 2021-03-05

**Authors:** Shelby L. Reimer, Daniel R. Beniac, Shannon L. Hiebert, Timothy F. Booth, Patrick M. Chong, Garrett R. Westmacott, George G. Zhanel, Denice C. Bay

**Affiliations:** ^1^Department of Medical Microbiology and Infectious Diseases, University of Manitoba, Winnipeg, MB, Canada; ^2^National Microbiology Laboratory, Public Health Agency of Canada, Winnipeg, MB, Canada

**Keywords:** outer membrane vesicles, *Escherichia coli*, ultracentrifugation, ultradiafiltration, hypervesiculation, Tol-Pal system, nanoparticle tracking analysis, LC-MS/MS

## Abstract

Outer membrane vesicles (OMVs) produced by Gram-negative bacteria are mediators of cell survival and pathogenesis by facilitating virulence factor dissemination and resistance to antimicrobials. Studies of OMV properties often focus on hypervesiculating *Escherichia coli* mutants that have increased OMV production when compared to their corresponding wild-type (WT) strains. Currently, two conventional techniques, ultracentrifugation (UC) and ultradiafiltration (UF), are used interchangeably to isolate OMVs, however, there is concern that each technique may inadvertently alter the properties of isolated OMVs during study. To address this concern, we compared two OMV isolation methods, UC and UF, with respect to final OMV quantities, size distributions, and morphologies using a hypervesiculating *Escherichia coli* K-12 Δ*tolA* mutant. Nanoparticle tracking analysis (NTA) indicated that UC techniques result in lower vesicle yields compared to UF. However, UF permitted isolation of OMVs with smaller average sizes than UC, highlighting a potential OMV isolation size bias by each technique. Cryo-transmission electron microscopy (cryo-TEM) visualization of isolated OMVs revealed distinct morphological differences between WT and Δ*tolA* OMVs, where Δ*tolA* OMVs isolated by either UC or UF method possessed a greater proportion of OMVs with two or more membranes. Proteomic OMV analysis of WT and Δ*tolA* OMVs confirmed that Δ*tolA* enhances inner plasma membrane carryover in multi-lamellar OMVs. This study demonstrates that UC and UF are useful techniques for OMV isolation, where UF may be preferable due to faster isolation, higher OMV yields and enrichment of smaller sized vesicles.

## Introduction

Bacterial outer membrane vesicles (OMVs) are spherical membrane structures typically ranging from 20-200 nm in diameter that are released from the outer membrane (OM) of Gram-negative bacteria into the extracellular milieu (Mashburn-Warren et al., 2008; Schwechheimer and Kuehn, 2015). OMVs are constitutively released from bacteria, in culture and during host infection, where they transport cargo such as toxins, virulence factors, autolysins, DNA and RNA (Jan, 2017; Cecil et al., 2019). OMVs play a critical role in promoting bacterial survival in stressful conditions, intercellular communication between bacteria, and by modulating host-pathogen interactions (Mashburn-Warren et al., 2008; Kulkarni and Jagannadham, 2014; Schwechheimer and Kuehn, 2015; Jan, 2017; Cecil et al., 2019). For example, OMVs from a variety of bacterial species can modulate the host immune response by activating immune cells and promoting cytokine secretion (Cecil et al., 2017), by delivering cytotoxic factors that induce apoptosis after internalization into host cells (Chmiela et al., 2018), and by secreting substances that damage surrounding tissues (O’Donoghue and Krachler, 2016; Cecil et al., 2019). OMVs have been proposed as specialized delivery vehicles, with their lipid bilayer topology ideal for transporting therapeutics to specific host cells (O’Donoghue and Krachler, 2016; Cecil et al., 2019). They have been incorporated into vaccine preparations due to their immunogenicity and ability to display antigens without the accompanying risk posed by metabolically active bacterial cells (Cecil et al., 2019). However, an important drawback for these applications is the low yield of vesicles that can be recovered from *in vitro* culture supernatants.

Previous studies seeking to identify genes associated with higher OMV production by *Escherichia coli* have involved gene knockout and gene disruption screens (McBroom et al., 2006; Kulp et al., 2015). Based on these studies, it was shown that mutations in certain membrane protein genes altered the OM architecture of *E. coli*, leading to hypervesiculation phenotypes with increased OMV production (Bernadac et al., 1998; Moon et al., 2012; Kulp et al., 2015; Turner et al., 2015; Pérez-Cruz et al., 2016). An important example is the *E. coli* Tol-Pal proteins, which are encoded within a seven gene cluster (*ybgC, tolQ, tolR, tolA, tolB, pal*, and *cpoB*) expressed from promoters upstream of *ybgC* and *tolB*; these proteins are vital for membrane maintenance and integrity of Gram-negative bacteria (Webster, 1991; Vianney et al., 1996; Bernadac et al., 1998; Lazzaroni et al., 1999; Lloubès et al., 2001; Cascales et al., 2002). The Tol-Pal system is composed of five interacting proteins that form a *trans*-membrane protein complex in the periplasmic space and associate with OmpA and Lpp in the OM (Lloubès et al., 2001). Mutations in any of the Tol-Pal genes can confer defects in the OM that lead to the activation of regulatory cascades responsible for extra-cytoplasmic stress responses, hypersensitivity to drugs and detergents, release of periplasmic proteins into the medium, and increased formation of OMVs (Bernadac et al., 1998; Lloubès et al., 2001; Vinés Marolda et al., 2005; Turner et al., 2015; Pérez-Cruz et al., 2016). Most recently, Δ*tolB* mutants of aquatic bacteria *Buttiauxella agrestis* and other Gram-negative species demonstrated that the loss of TolB enhanced the formation of multi-lamellar/multi-vesicular OMVs, referred to as M-OMVs (Takaki et al., 2020). As a result, the Tol-Pal system is of particular interest and importance to researchers seeking to better understand *E. coli* OMV morphology, formation and production.

One of the main limitations involved in studying OMVs is the challenging isolation and purification methods required to obtain sufficient quantities of these small vesicular structures. Techniques cited by most authors include ultracentrifugation and ultrafiltration (Horstman and Kuehn, 2000; Wai et al., 2003; Lee et al., 2007; Chutkan et al., 2013). It is important to note that the isolation method may affect an OMV’s morphology and total yield, promote aggregation of OMVs, and/or collect lipoproteins and other unwanted cell debris (Witwer et al., 2013; Yuana et al., 2014). Thus, an ideal OMV isolation method should provide high OMV yields without damaging vesicles for downstream experimental analyses or biotechnological applications. At the present time, comparative studies of OMV isolation methods and OMV quantifications are lacking, but both are important for improved in-depth analyses of OMVs.

The goal of our study was to evaluate two of the most commonly used OMV isolation techniques, ultracentrifugation (UC) and ultradiafiltration (UF) with an *E. coli* K-12 BW25113 strain (WT) and JW0729 (Δ*tolA*), a mutant containing a single-gene deletion of TolA component in the Tol-Pal system. Δ*tolA* was selected for comparison as previous studies (Bernadac et al., 1998; McBroom et al., 2006) identified that this deletion mutant confers a hypervesiculating phenotype when compared to the WT *E. coli* strain. In our study, we compared OMVs isolated from both strains grown under identical growth conditions to assess the yield and size of vesicles with nanoparticle tracking analysis (NTA). NTA rapidly detects nanoparticles in solution by combining laser light scattering microscopy with a charge-coupled device camera to visualize particles. These detected nanoparticles are assessed with software to relate the rate of particle movement by Brownian motion to its particle size in nm according to the Stokes–Einstein equation (Filipe et al., 2010; Gardiner et al., 2013; Gerritzen et al., 2017). Using NTA is advantageous to other OMV quantification methods as it allows for direct measurement of polydisperse samples, while the flow mode allows a large number of particles to be measured in a small timeframe, resulting in more accurate measurements with less variance (Filipe et al., 2010; Gerritzen et al., 2017). Cryo-transmission electron microscopy (cryo-TEM) analysis was used to visually determine OMV morphology and verify OMV size and total quantity produced by each isolation technique. Comparing OMV isolations from a WT strain as well as a hypervesiculating Δ*tolA* strain allowed us to explore the limits of each technique. We also employed Nano-LC/MS/MS proteomic analysis to compare the protein compositions of WT and Δ*tolA* vesicles. The outcome of this analysis revealed that UC and UF methods are similar with the exception of OMV minimal size limits. It allowed us to provide the first in-depth characterization of Δ*tolA* mutant OMVs, which revealed not only an increase in Δ*tolA* OMV quantity but also Δ*tolA* vesicles with two (outer-inner membrane vesicles; O-IMVs) or more membranes (multi-lamellar outer membrane vesicles; M-OMVs, grouped outer membrane vesicles; G-OMVs) by cryo-TEM visualization. Proteomic analysis of WT and Δ*tolA* OMVs demonstrated that Δ*tolA* OMVs possess more inner membrane (IM), periplasmic, and cytoplasmic proteins than WT, indicating that the loss of TolA may decrease linkages between the outer and inner membranes and result in the formation of these unique vesicle morphologies, similar to phenotypes recently described in Δ*tolB* and Δ*tolR* mutants (Pérez-Cruz et al., 2016; Takaki et al., 2020).

## Results

### Δ*tolA* Produces Significantly More Vesicles Than WT

**FIGURE 1:**
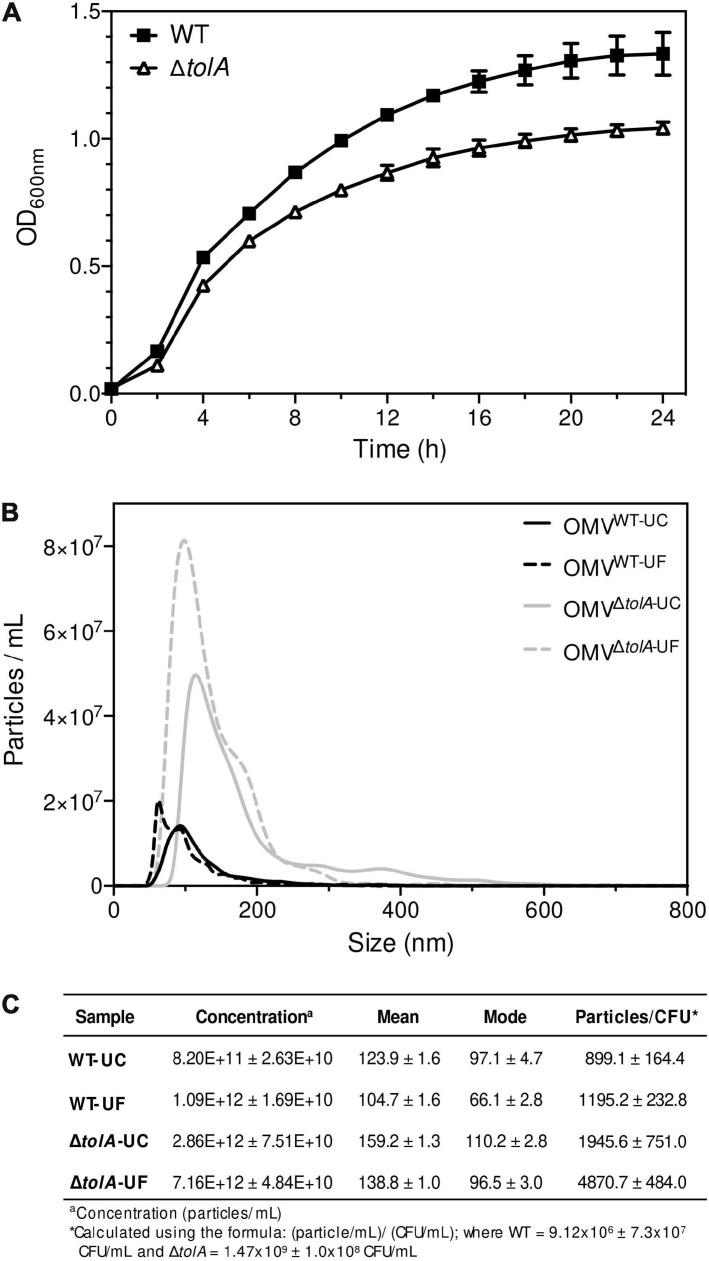
Growth curve analysis of *Escherichia coli* wild-type and Δ*tolA* and NTA measurements of their isolated OMVs. **(A)** Growth curves of BW25113 (WT) and JW0729 (Δ*tolA*) *E. coli* strains. OD_600 *nm*_ measurements (*y*-axis) are shown over time (h; *x*-axis) for WT (squares) and Δ*tolA* (triangles) strains grown in LB broth at 37°C plates every 2 h for 24 h at 37°C. Growth curves represent the mean of two biological samples measured in triplicate (*n* = 6), where error bars represent the standard deviation of mean value. WT and Δ*tolA* growth were significantly different for each time point except 0 h according to a Mann–Whitney *U* test (*p* < 0.05). **(B)** OMVs isolated from WT and Δ*tolA* strains using either UC or UF methods (refer to panel legend) were diluted 1,000-fold and measured using a Nanosight NS500. All data represent the mean of triplicate experiments ± standard error. **(C)** A summary of NTA data collected, listing WT and Δ*tolA* mean vesicle size (nm), mode size (nm), concentration (particles/mL), and particles/CFU for each isolation method.

The primary aim of this study was to compare two of the most commonly used UC and UF OMV isolation methods and in doing so, provide an opportunity to examine OMV production differences between an *E. coli* K-12 BW25113 (WT) strain and its hypervesiculating gene deletion mutant Δ*tolA* (JW0729). Prior to UC and UF OMV isolations, we wanted to ensure that OMV formation from WT and Δ*tolA* was proportional to the total quantity of cells grown in culture; this measurement was important to account for potential cell titer differences caused by growth rate differences between the mutant and WT. To accomplish this, we measured growth curves of each strain prior to OMV isolation (Figure 1A). WT and Δ*tolA* growth rates were significantly different (*p* < 0.05) in optical density at 600 nm (OD_600 *nm*_) unit values for all time points, and the maximum OD_600 *nm*_ units for WT was 1.11 ± 0.03 and Δ*tolA* was 0.98 ± 0.02 after 24 h (Figure 1A). Due to lower OD_600 *nm*_ values of Δ*tolA*, we calculated OMV production yields based on total cells in colony forming units (CFU)/mL from OD_600 *nm*_ measurements of each culture. This allowed a more accurate compare comparison of WT and Δ*tolA* OMV formation and quantity differences by UC and UF methods, and these values are listed in Figure 1C. For all comparisons made between UC and UF, a single large-scale bacterial culture was grown, and equally divided for UC and UF OMV isolations in order to minimize differences in OMV populations caused by batch growth effects.

After factoring in the cell growth differences of each strain, we compared differences in OMV production by each strain when isolated by UC and UF methods using NTA (Figures 1B,C). NTA demonstrated statistically significant differences between WT and Δ*tolA* strains with respect to vesicle production. Both UC and UF methods confirmed greater Δ*tolA* OMVs quantities when compared to the WT based on particle/CFU calculations, corroborating previous studies on OMV production in Tol-Pal mutants (Bernadac et al., 1998; McBroom et al., 2006; Kulp et al., 2015; Pérez-Cruz et al., 2016; Takaki et al., 2020). Specifically, UC had a 2.2 (±0.45)-fold increase in Δ*tolA* OMVs when compared to WT and UF had a 4.1 (±0.41)-fold increase in Δ*tolA* OMVs compared to WT. NTA results also showed fewer WT and Δ*tolA* OMVs were recovered by UC methods when compared to UF (WT; 24.8% reduction, Δ*tolA* 60.0% reduction; Figures 1B,C). Hence, isolating OMVs using UC and UF methods confirmed that the Δ*tolA* strain hypervesiculates when compared to WT grown under the same conditions, but UF methods recover more OMVs as compared to UC.

### OMVs Isolated by UC and UF Show no Differences in OmpA Abundance

**FIGURE 2:**
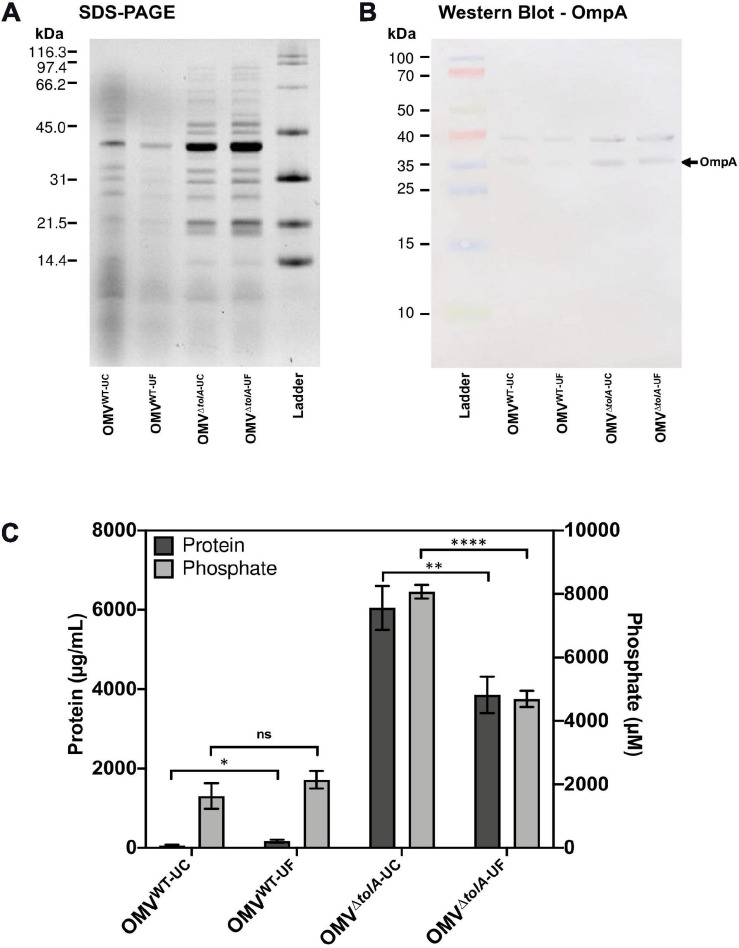
Protein and phosphorous profiles of isolated OMVs. **(A)** 12% acrylamide Tricine SDS-PAGE gel image of UC and UF OMV isolations for WT and Δ*tolA E. coli* strains detected with TCE. **(B)** Western blot of isolated OMV samples from WT and Δ*tolA E. coli* strains detected with anti-OmpA antibodies (1:25,000). **(C)** OMV sample total protein and total phosphorous content for each OMV isolation method. Values were obtained by BCA assay (protein) or malachite green assay (phosphate) and adjusted based on dilution factor and OD_600 *nm*_ of the original culture. All data represent the mean of triplicate measurements, and error bars represent standard deviation. Significant differences between WT and Δ*tolA* samples or between UC and UF samples were determined using the Mann–Whitney *U* test at *p*-values of <0.05 (*), <0.005 (**), <0.00005 (****).

To compare the differences in OMV content that may occur due to the isolation methods themselves, we performed Tricine sodium dodecyl sulfate-polyacrylamide gel electrophoresis (Tricine SDS-PAGE) analysis to determine if any OMV protein content was noticeably altered (Figure 2A). There were no significant differences in densitometry of stained protein bands between UC and UF OMV preparations for either strain. To determine if there were differences in key OM porins, Western blot analysis was performed to compare OmpA content ratios in OMVs. OmpA porin proteins are abundant and located in the OM, making them a reliable OMV detection marker (Bielaszewska et al., 2017). Based on this analysis, both UC and UF methods showed enrichment of OmpA in Δ*tolA* OMVs compared to WT based on net Δ*tolA* OmpA pixel density/WT OmpA pixel density (UC; 1.27, UF; 1.85, Figure 2B), and no significant differences in OmpA protein abundance between UC and UF-isolated OMV samples for either Δ*tolA* or WT (Figure 2B). This suggests that OmpA proteins present in OMVs can be accurately detected in both UC and UF isolation methods, indicating that the isolation method does not influence OmpA protein detection accuracy. This result indicates that OmpA could be a reliable detection marker for OMV production, as the ratio of OmpA present in our WT and Δ*tolA* OMV samples were comparable to OMV concentration ratios of WT and Δ*tolA* from NTA analysis.

### UC and UF Isolation Methods Differ in Concentration and Size of Recovered OMVs

In addition to NTA analysis, UC and UF OMVs isolated from WT and Δ*tolA* cultures were quantified by total protein bicinchoninic acid (BCA), and total lipid phosphorous (Malachite Green) assays to determine if UC and UF methods influence total protein or lipid phosphate contents in OMVs. As noted in our OMV OmpA protein detection experiments above, we wanted to determine if total protein and lipid OMV contents were altered specifically by each isolation method, as each method may differentially shift protein and lipid content carry-over. Total protein and lipid assays are routinely used to enumerate OMVs and to quantify protein–lipid content ratios of OMVs (McBroom et al., 2006; Orench-Rivera and Kuehn, 2016; Roier et al., 2016). Discordant results for WT and Δ*tolA* total protein and total phosphate were noted for UC and UF methods (Figure 2C). Significantly higher protein (*p* < 0.005) and phosphate (*p* < 0.00005) concentrations were detected in the UC-isolated Δ*tolA* sample as compared to Δ*tolA* OMVs isolated by UF, indicating greater protein and phosphate content in OMVs from these preparations (Figure 2C). The UF-isolated WT samples had significantly higher protein concentration (*p* < 0.05) than the UC-isolated WT, but no significant concentration differences between UC and UF WT isolations for phosphate concentration (Figure 2C). When considered with NTA data (Figures 1B,C), these results suggest that UF OMV isolation may enhance vesicle isolation yields as compared to UC but may also affect the WT and Δ*tolA* total protein and phosphate content.

Next, we determined the average vesicle sizes of WT and Δ*tolA* by NTA to determine if either method significantly altered the size of OMVs recovered (Figure 1C). The average size of UC-isolated OMVs was 123.9 ± 1.6 nm [mean ± standard error of the mean (SEM)] in dia for WT and 159.2 ± 1.3 nm dia for Δ*tolA*. UF-isolated OMVs had smaller average sizes of WT and Δ*tolA* vesicles at 104.7 ± 1.6 nm dia and 138.8 ± 1.0 nm dia, respectively. Thus, OMVs formed by the Δ*tolA* strain were larger in size as compared to the WT control by both methods (Figure 1B, *p* < 0.0001). When analyzing OMV particle size distributions, we also noticed that all UF-isolated OMV samples had a larger proportion of smaller sized vesicles when compared to vesicles isolated by UC which was enriched with larger sized vesicles. OMVs with diameter sizes between 0 and 100 nm were greatly enriched in UF isolations (WT UF; 74.3%, Δ*tolA* UF; 50.4%) as compared to UC (WT UC; 58.1%, Δ*tolA* UC; 38.3%; Supplementary Figure 1A). The opposite was true for UC-isolated vesicles, which had vesicles predominating at larger sizes ranging between 200 and 550 nm (Supplementary Figure 1B). OMVs with diameters over 100 nm corresponded to 41.9% of the total OMVs in UC-isolated WT samples and 61.7% in UC-isolated Δ*tolA* samples, whereas this range was 25.7% in UF-isolated WT samples and 49.6% in UF-isolated Δ*tolA* samples. These findings indicate that a size isolation bias exists for each method, where UF enriches for smaller sized particles when compared to the UC method.

### Cryo-TEM of Δ*tolA* OMVs Reveals Distinct Morphological Differences From WT OMVs

**FIGURE 3:**
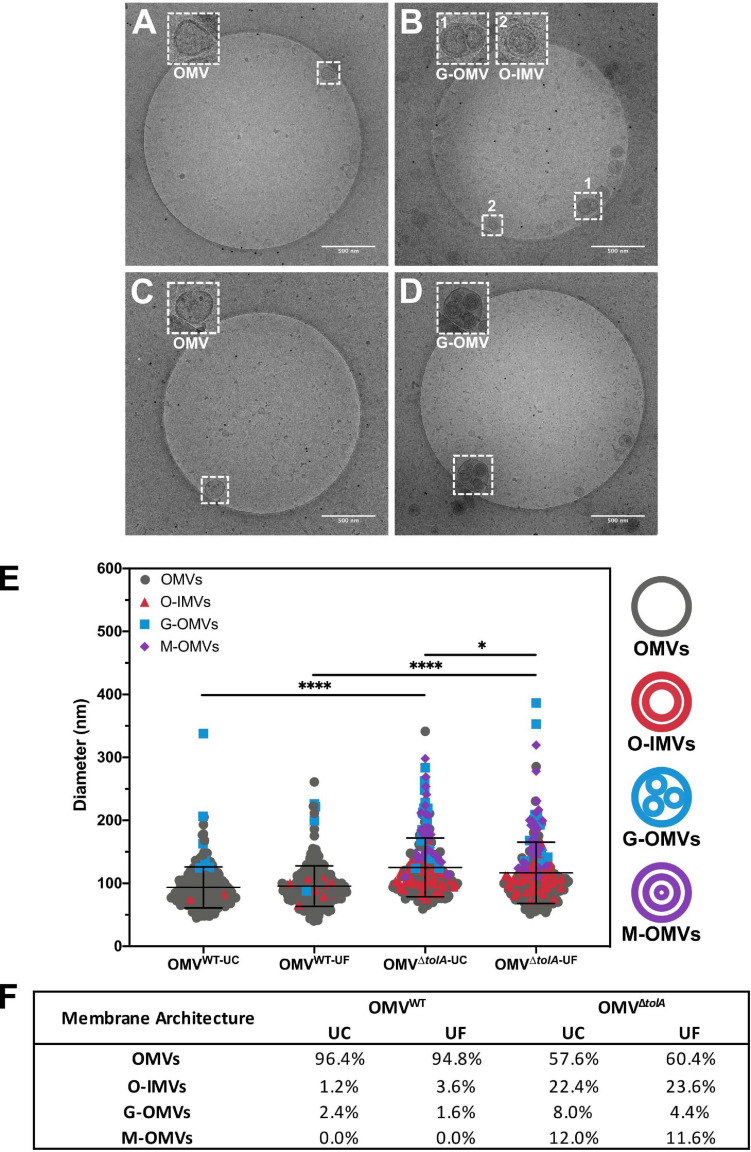
Cryo-TEM images of vesicle types in WT and Δ*tolA* strains. Representative cryo-TEM images at 14,500× magnification of WT OMVs isolated by UC **(A)**, WT OMVs isolated by UF **(B)**, Δ*tolA* OMVs isolated by UC **(C)**, Δ*tolA* OMVs isolated by UF **(D)**. In each panel, enlarged images of representative OMVs frequently observed (based on table values shown in panel **F**) in WT and Δ*tolA* are highlighted as inset panel images in dashed boxes in the upper left-hand corner. Conventional OMVs as well as vesicles with two membranes (O-IMVs) and multi-layered vesicles (M-OMVs, G-OMVs) are shown in these inset images in panels **(A–D)**. **(E)** Scatter plot summaries of vesicle diameters measured from cryo-TEM images of OMVs isolated from the WT and Δ*tolA* strains using either ultracentrifugation (UC) or ultradiafiltration (UF) at *N* = 250 vesicles/strain. Lines on each plot represent the mean value, and error bars represent standard deviations. Diameters of vesicles from cryo-TEM images were measured using ImageJ particle analysis software. Shape and color of data points represent the types of vesicle visualized (refer to in panel legend). Significance was determined using Mann–Whitney *U* test ^∗^*p* < 0.01, ^****^p < 0.0001, *N* = 250 vesicles). **(F)** Summary table of membrane vesicle architectures manually identified from cryo-TEM images using ImageJ. Values listed in the table represent the percent total number of vesicles manually assessed representing each shape/architecture type (*N* = 250 vesicles/strain).

OMV morphology analysis of each vesicle isolation method and strain type was performed using cryo-TEM analysis to establish any vesicle size and heterogeneity alterations. Statistical analysis of OMV measurements from cryo-TEM photomicrographs of WT and Δ*tolA* strains was performed, where representative examples are shown in Figures 3A–D, and revealed significant differences in vesicle size as summarized in Figure 3E. Measurements from cryo-TEM vesicle images identified a range of OMV sizes (40–400 nm dia) for each strain and methodology used (Figure 3E), supporting our NTA findings (Figure 1B). However, based this image analysis, all OMVs isolated by either UC or UF had a smaller size distribution range when compared to the same preparations analyzed by NTA (IQR; WT UC; 72.3–107.8 nm, WT UF; 75.7–107.7 nm, Δ*tolA* UC; 93.1–141.4 nm, Δ*tolA* UF; 86.2–133.7 nm). Additionally, cryo-TEM imaged vesicle diameters of Δ*tolA* isolated by UC and UF methods demonstrated significant differences in size, where average vesicle size of UC-isolated Δ*tolA* OMVs was 125.2 nm and UF-isolated Δ*tolA* OMVs was 116.7 nm (*p* = 0.0097; Figure 3C). Cryo-TEM average measurements of WT OMV diameters from either isolation method were not significantly different (UC; 93.55 nm, UF; 95.58 nm, *p* = 0.3123; Figure 3C). Hence, NTA and cryo-TEM measurements are generally in agreement with respect to UC and UF OMV size ranges and size averages, but when comparing vesicle size distributions by NTA and cryo-TEM techniques, cryo-TEM measurements suggest smaller diameter vesicle sizes and averages for both WT and Δ*tolA* by both isolation techniques. This disparity is likely due to differences in the number of vesicles counted by each method, indicating that NTA may be more precise due to the quantity and range of particle sizes that are accurately measurable.

Lastly, cryo-TEM highlighted stark differences between WT and Δ*tolA* OMVs with respect to their overall morphology. WT OMV morphologies were characteristic of previously described OMVs by either UC or UF methods (Koning et al., 2013; Pérez-Cruz et al., 2016; Thoma et al., 2018). Based on our cryo-TEM images, nearly all WT OMVs had a single membrane, presumably composed of the OM bilayer (Figures 3A,C). In contrast, the Δ*tolA* mutant had OMVs with variable single and multi-membranous structures when isolated by either UC or UF technique (Figures 3B,D). The Δ*tolA* strain had a high proportion of OMVs with two or more membranes by both methods (UC; 39.6, UF; 42.4%; Figure 3F), which included double-bilayer outer-inner OMVs (O-IMVs), multi-layered vesicles (M-OMVs) (≥3 layers), as well as grouped encapsulated OMVs (G-OMVs) surrounded by a larger extramembrane layer (Figure 3B). These altered vesicle morphologies produced by the Δ*tolA* mutant likely account for the larger average sized vesicles detected by NTA. Δ*tolA* vesicles with multiple membranes had a significantly larger average size (UC; 146.7 nm, UF; 143.2 nm) than WT OMVs when measured by cryo-TEM (UC; 110.5 nm, UF; 98.5 nm; Figure 3D). Taken altogether with the results from total protein/total phosphorous, NTA size distributions, and our cryo-TEM vesicle morphology analyses, we can state that *tolA* mutations considerably alter OMV formation and morphology. These analyses also reveal that neither UC or UF significantly altered the recoverable amount of WT and Δ*tolA* OMV content or morphology, highlighting both as useful OMV isolation techniques.

### Proteomic Analysis Confirms IM Proteins in Δ*tolA* OMVs Which Were Absent From the WT

**FIGURE 4:**
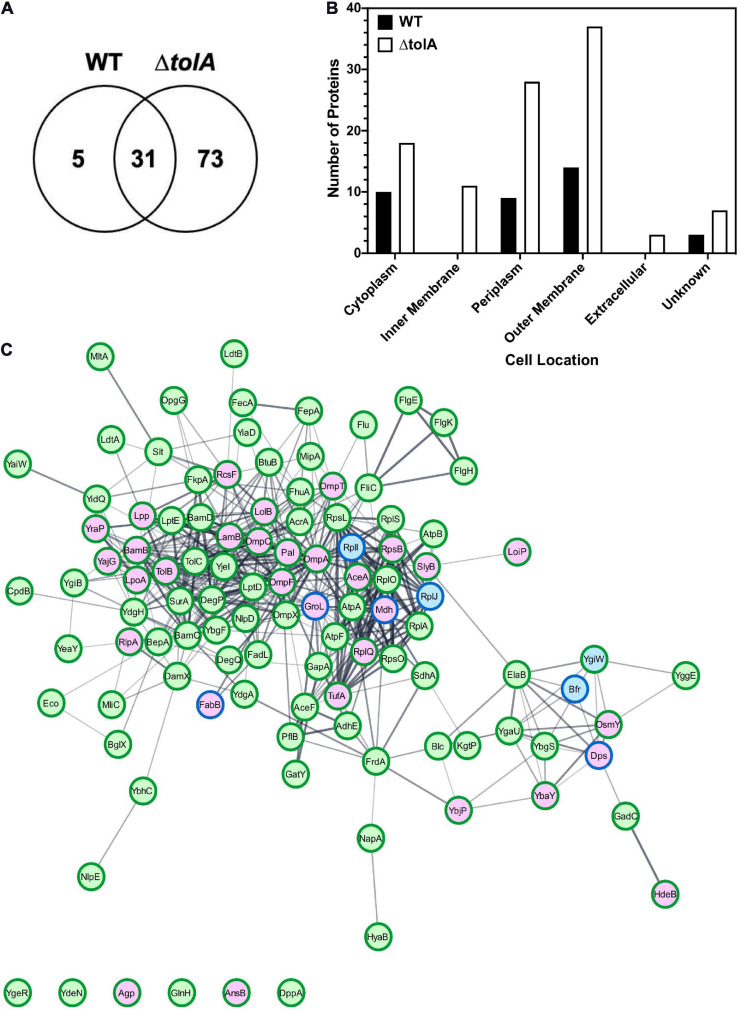
Proteomic characterization of WT and Δ*tolA* vesicles isolated by UF. **(A)** Venn-diagram of identified proteins showing overlap between WT and Δ*tolA* vesicle samples. **(B)** Cellular localization of identified proteins from WT (black) and Δ*tolA* (white) vesicle samples. **(C)** String network of interacting proteins from WT (blue), Δ*tolA* (green), or both (purple) vesicle samples. Border colors represent protein abundance, with dark green borders denoting upregulation of the protein in the Δ*tolA* sample, and dark blue borders denoting upregulation of the protein in the WT sample. The network diagram was generated using the StringApp v1.5.0 in Cytoscape v.3.8.0 (Doncheva et al., 2019). All data used in this analysis are summarized in Table 1.

In an effort to further investigate the membrane contents of both WT and Δ*tolA* OMVs we used a proteomic approach to identify altered or unique OMV proteins (Figure 4). We performed in-depth nano-LC MS/MS analysis on WT and Δ*tolA* UF OMV preparations only, since these preparations produced greater yields of OMVs, at size ranges also present in UC methods. Our preliminary analyses of WT and Δ*tolA* OMVs proteomes including SDS-PAGE (Figure 2) did not reveal any significant differences in UC or UF proteins, which is not surprising given these OMVs were isolated from the same starting cultures. A total of 109 proteins were identified in this UF OMV proteomic analysis, where only 31 proteins were detected in both the WT and the *tolA* mutant (Figure 4A and Table 1). Only 5 proteins were exclusively over-accumulated in WT OMVs, whereas 73 proteins were exclusively enriched in Δ*tolA* OMVs (Figure 4A and Table 1). This initial analysis indicates that the mutant has a larger number of proteins sequestered in its vesicles as compared to WT, as we expected from its M-OMV morphology visualized in cryo-TEM images.

**TABLE 1:** List of proteins identified by proteomic analysis in WT and Δ*tolA* OMVs isolated by UF.

UniProtKB AC/ID	Gene	Protein	Protein detection in strain(s)	Fisher’s exact test (*p*-value): (*p* < 0.05)	Fold change by sample	Quantitative profile
**CYTOPLASM**						
P0A9G6	*aceA*	Isocitrate lyase	BOTH	<0.00010	1.1	Increased in ΔtolA
P06959	*aceF*	Dihydrolipoyllysine-residue acetyltransferase component of pyruvate dehydrogenase complex	Δ*tolA*	0.015	INF	Increased in ΔtolA
P0A9Q7	*adhE*	Aldehyde-alcohol dehydrogenase	Δ*tolA*	<0.00010	78	Increased in ΔtolA
P0ABB0	*atpA*	ATP synthase subunit alpha	Δ*tolA*	0.011	INF	Increased in ΔtolA
P0ABD3	*bfr*	Bacterioferritin	WT	<0.00010	0	Increased in WT
P0ABT2	*dps*	DNA protection during starvation protein	BOTH	<0.00010	0.3	Increased in WT
P0A953	*fabB*	3-oxoacyl-[acyl-carrier-protein] synthase 1	BOTH	<0.00010	0.8	Increased in WT
P0A9B2	*gapA*	Glyceraldehyde-3-phosphate dehydrogenase A	Δ*tolA*	0.00094	INF	Increased in ΔtolA
P0C8J6	*gatY*	D-tagatose-1,6-bisphosphate aldolase subunit GatY	Δ*tolA*	<0.00010	INF	Increased in ΔtolA
P0A6F5	*groL*	60 kDa chaperonin 1	BOTH	<0.00010	0.4	Increased in WT
P09373	*pflB*	Formate acetyltransferase 1	Δ*tolA*	0.022	INF	Increased in ΔtolA
P0A7L0	*rplA*	50S ribosomal protein L1	Δ*tolA*	0.00023	INF	Increased in ΔtolA
P0A7R1	*rplI*	50S ribosomal protein L9 OS = Escherichia coli	WT	0.00065	0	Increased in WT
P0A7J3	*rplJ*	50S ribosomal protein L10	WT	0.0001	0	Increased in WT
P02413	*rplO*	50S ribosomal protein L15	ΔtolA	0.011	INF	Increased in ΔtolA
P0AG44	*rplQ*	50S ribosomal protein L17	BOTH	0.011	2.4	Increased in ΔtolA
P0A7K6	*rplS*	50S ribosomal protein L19	Δ*tolA*	0.022	INF	Increased in ΔtolA
P0A7V0	*rpsB*	30S ribosomal protein S2	BOTH	0.011	2.5	Increased in ΔtolA
P0A7S3	*rpsL*	30S ribosomal protein S12	Δ*tolA*	0.0019	INF	Increased in ΔtolA
P0ADZ4	*rpsO*	30S ribosomal protein S15	Δ*tolA*	0.0091	INF	Increased in ΔtolA
P0CE47	*tufA*	Elongation factor Tu 1	BOTH	<0.00010	2	Increased in ΔtolA
**EXTRACELLULAR**						
P75937	*flgE*	Flagellar hook protein FlgE	Δ*tolA*	<0.00010	INF	Increased in ΔtolA
P33235	*flgK*	Flagellar hook-associated protein 1	Δ*tolA*	0.022	INF	Increased in ΔtolA
P04949	*fliC*	Flagellin	Δ*tolA*	<0.00010	INF	Increased in ΔtolA
**INNER MEMBRANE**						
P0AE06	*acrA*	Multidrug efflux pump subunit AcrA	Δ*tolA*	0.015	INF	Increased in ΔtolA
P0AB98	*atpD*	ATP synthase subunit beta	Δ*tolA*	0.026	INF	Increased in ΔtolA
P0ABA0	*atpF*	ATP synthase subunit b	Δ*tolA*	0.0091	INF	Increased in ΔtolA
P11557	*damX*	Cell division protein DamX	Δ*tolA*	0.031	INF	Increased in ΔtolA
P0AEH5	*elaB*	Protein ElaB	Δ*tolA*	<0.00010	INF	Increased in ΔtolA
P00363	*frdA*	Fumarate reductase flavoprotein subunit	Δ*tolA*	0.044	INF	Increased in ΔtolA
P63235	*gadC*	Probable glutamate/gamma-aminobutyrate antiporter	Δ*tolA*	0.00039	INF	Increased in ΔtolA
P0ACD8	*mbhL*	Hydrogenase-1 large chain	Δ*tolA*	0.0027	INF	Increased in ΔtolA
P0AEX3	*kgtP*	Alpha-ketoglutarate permease	Δ*tolA*	0.031	INF	Increased in ΔtolA
P0AC41	*sdhA*	Succinate dehydrogenase flavoprotein subunit	Δ*tolA*	0.0013	INF	Increased in ΔtolA
P77804	*ydgA*	Protein YdgA	Δ*tolA*	0.015	INF	Increased in ΔtolA
**OUTER MEMBRANE**						
P77774	*bamB*	Outer membrane protein assembly factor BamB	BOTH	<0.00010	23	Increased in ΔtolA
P0A903	bamC	Outer membrane protein assembly factor BamC	Δ*tolA*	<0.00010	INF	Increased in ΔtolA
P0AC02	*bamD*	Outer membrane protein assembly factor BamD	Δ*tolA*	<0.00010	INF	Increased in ΔtolA
P0A901	*blc*	Outer membrane lipoprotein Blc	Δ*tolA*	0.013	INF	Increased in ΔtolA
P06129	*btuB*	Vitamin B12 transporter BtuB	Δ*tolA*	0.0012	31	Increased in ΔtolA
P10384	*fadL*	Long-chain fatty acid transport protein	Δ*tolA*	<0.00010	79	Increased in ΔtolA
P13036	*fecA*	Fe(3+) dicitrate transport protein FecA	Δ*tolA*	<0.00010	INF	Increased in ΔtolA
P05825	*fepA*	Ferrienterobactin receptor	Δ*tolA*	0.00094	INF	Increased in ΔtolA
P06971	*fhuA*	Ferrichrome-iron receptor	Δ*tolA*	<0.00010	INF	Increased in ΔtolA
P0A6S0	*flgH*	Flagellar L-ring protein	Δ*tolA*	<0.00010	INF	Increased in ΔtolA
P39180	*flu*	Antigen 43	Δ*tolA*	<0.00010	41	Increased in ΔtolA
P02943	*lamB*	Maltoporin	BOTH	<0.00010	28	Increased in ΔtolA
P25894	*loiP*	Metalloprotease LoiP	BOTH	<0.00010	43	Increased in ΔtolA
P61320	*lolB*	Outer-membrane lipoprotein LolB	BOTH	<0.00010	35	Increased in ΔtolA
P45464	*lpoA*	Penicillin-binding protein activator LpoA	BOTH	<0.00010	19	Increased in ΔtolA
P69776	*lpp*	Major outer membrane prolipoprotein Lpp	BOTH	<0.00010	3.3	Increased in ΔtolA
P31554	*lptD*	LPS-assembly protein LptD	Δ*tolA*	<0.00010	INF	Increased in ΔtolA
P0ADC1	*lptE*	LPS-assembly lipoprotein LptE	Δ*tolA*	<0.00010	INF	Increased in ΔtolA
P0A908	*mipA*	MltA-interacting protein	Δ*tolA*	<0.00010	28	Increased in ΔtolA
P28224	*mliC*	Membrane-bound lysozyme inhibitor of C-type lysozyme	Δ*tolA*	<0.00010	INF	Increased in ΔtolA
P0A935	*mltA*	Membrane-bound lytic murein transglycosylase A	Δ*tolA*	0.026	INF	Increased in ΔtolA
P0ADA3	*nlpD*	Murein hydrolase activator NlpD	Δ*tolA*	<0.00010	89	Increased in ΔtolA
P40710	*nlpE*	Lipoprotein NlpE	Δ*tolA*	0.028	2.9	Increased in ΔtolA
P0A910	*ompA*	Outer membrane protein A	BOTH	<0.00010	2.2	Increased in ΔtolA
P06996	*ompC*	Outer membrane protein C	BOTH	<0.00010	3.9	Increased in ΔtolA
P02931	*ompF*	Outer membrane protein F	BOTH	<0.00010	21	Increased in ΔtolA
P09169	*ompT*	Protease 7	BOTH	0.00021	14	Increased in ΔtolA
P0A917	*ompX*	Outer membrane protein X	Δ*tolA*	<0.00010	INF	Increased in ΔtolA
P0A912	*pal*	Peptidoglycan-associated lipoprotein	BOTH	0.023	4	Increased in ΔtolA
P69411	*rcsF*	Outer membrane lipoprotein RcsF	BOTH	0.00014	14	Increased in ΔtolA
P10100	*rlpA*	Endolytic peptidoglycan transglycosylase RlpA	BOTH	0.0014	9.7	Increased in ΔtolA
P0A905	*slyB*	Outer membrane lipoprotein SlyB	BOTH	<0.00010	2.2	Increased in ΔtolA
P02930	*tolC*	Outer membrane protein TolC	Δ*tolA*	<0.00010	INF	Increased in ΔtolA
P46130	*ybhC*	Putative acyl-CoA thioester hydrolase YbhC	Δ*tolA*	0.0017	20	Increased in ΔtolA
P0AA91	*yeaY*	Uncharacterized lipoprotein YeaY	Δ*tolA*	<0.00010	INF	Increased in ΔtolA
Q46798	*ygeR*	Uncharacterized lipoprotein YgeR	Δ*tolA*	<0.00010	INF	Increased in ΔtolA
P37665	*yiaD*	Probable lipoprotein YiaD	Δ*tolA*	0.0017	30	Increased in ΔtolA
**PERIPLASM**						
P19926	*agp*	Glucose-1-phosphatase	BOTH	0.032	8.9	Increased in ΔtolA
P00805	*ansB*	L-asparaginase 2	BOTH	<0.00010	22	Increased in ΔtolA
P66948	*bepA*	Beta-barrel assembly-enhancing protease	Δ*tolA*	0.015	INF	Increased in ΔtolA
P33363	*bglX*	Periplasmic beta-glucosidase	Δ*tolA*	0.0023	INF	Increased in ΔtolA
P08331	*cpdB*	2′,3′-cyclic-nucleotide 2′-phosphodiesterase/3′-nucleotidase	Δ*tolA*	0.0091	INF	Increased in ΔtolA
P45955	*cpoB*	Cell division coordinator CpoB	Δ*tolA*	<0.00010	64	Increased in ΔtolA
P0C0V0	*degP*	Periplasmic serine endoprotease DegP	Δ*tolA*	<0.00010	INF	Increased in ΔtolA
P39099	*degQ*	Periplasmic pH-dependent serine endoprotease DegQ	Δ*tolA*	0.00094	INF	Increased in ΔtolA
P23847	*dppA*	Periplasmic dipeptide transport protein	Δ*tolA*	0.00025	19	Increased in ΔtolA
P23827	*eco*	Ecotin	Δ*tolA*	0.0045	INF	Increased in ΔtolA
P39176	*erfK*	Probable L,D-transpeptidase ErfK/SrfK	Δ*tolA*	0.0027	INF	Increased in ΔtolA
P45523	*fkpA*	FKBP-type peptidyl-prolyl *cis–trans* isomerase FkpA	Δ*tolA*	0.0061	20	Increased in ΔtolA
P0AEQ3	*glnH*	Glutamine-binding periplasmic protein	Δ*tolA*	0.04	19	Increased in ΔtolA
P0AET2	*hdeB*	Acid stress chaperone HdeB	BOTH	<0.00010	1.2	Increased in ΔtolA
P61889	*mdh*	Malate dehydrogenase	BOTH	<0.00010	0.8	Increased in WT
P33136	*mdoG*	Glucans biosynthesis protein G	Δ*tolA*	0.0091	INF	Increased in ΔtolA
P33937	*napA*	Periplasmic nitrate reductase	Δ*tolA*	0.00039	INF	Increased in ΔtolA
P0AFH8	*osmY*	Osmotically-inducible protein Y	BOTH	<0.00010	2.3	Increased in ΔtolA
P0AGC3	*slt*	Soluble lytic murein transglycosylase	Δ*tolA*	<0.00010	INF	Increased in ΔtolA
P0ABZ6	*surA*	Chaperone SurA	Δ*tolA*	0.0014	24	Increased in ΔtolA
P0A855	*tolB*	Protein TolB	BOTH	0.0057	7	Increased in ΔtolA
P77717	*ybaY*	Uncharacterized lipoprotein YbaY	BOTH	<0.00010	1.7	Increased in ΔtolA
P0AAV6	*ybgS*	Uncharacterized protein YbgS	Δ*tolA*	<0.00010	INF	Increased in ΔtolA
P0AAX8	*ybiS*	Probable L,D-transpeptidase YbiS	Δ*tolA*	0.009	12	Increased in ΔtolA
P77318	*ydeN*	Uncharacterized sulfatase YdeN	Δ*tolA*	0.0064	INF	Increased in ΔtolA
P0ADS6	*yggE*	Uncharacterized protein YggE	Δ*tolA*	<0.00010	INF	Increased in ΔtolA
P0ADT2	*ygiB*	UPF0441 protein YgiB	Δ*tolA*	0.044	INF	Increased in ΔtolA
P0ADU5	*ygiW*	Protein YgiW	WT	0.016	1.8	Increased in ΔtolA
P64596	*yraP*	Uncharacterized protein YraP	BOTH	0.039	9.1	Increased in ΔtolA
**UNKNOWN**						
P0ADE6	*kbp*	Potassium binding protein Kbp	ΔtolA	0.037	INF	Increased in ΔtolA
P77562	*yaiW*	Uncharacterized protein YaiW	ΔtolA	0.00028	INF	Increased in ΔtolA
P0ADA5	*yajG*	Uncharacterized lipoprotein YajG	BOTH	<0.00010	1.2	Increased in ΔtolA
P75818	*ybjP*	Uncharacterized lipoprotein YbjP	BOTH	0.017	9.5	Increased in ΔtolA
P76177	*ydgH*	Protein YdgH	Δ*tolA*	<0.00010	INF	Increased in ΔtolA
P0ADM4	*yidQ*	Uncharacterized protein YidQ	Δ*tolA*	0.0091	INF	Increased in ΔtolA
P0AF70	*yjeI*	Uncharacterized protein YjeI	Δ*tolA*	0.00013	29	Increased in ΔtolA

*AC, accession number; ID, identification number; INF, infinity value as the denominator in the fold change equation is zero.*

To determine the membrane location of proteins we detected in each strain’s OMV preparation, we annotated the identified proteins and predicted their subcellular localization using the pSORTb algorithm (Yu et al., 2010), as shown in Figure 4B. 61% of the proteins we identified in both strains were classified as either OM proteins (37 proteins) or periplasmic proteins (29 proteins), whereas a smaller proportion of proteins from the IM (10 proteins) and cytoplasm (21 proteins) were detected primarily in Δ*tolA* mutant vesicles (Figure 4B). Of the remaining 10% of subcellularly localized proteins, 3 were predicted to be secreted proteins, and 8 had an unknown localization. From this analysis, we noted that all proteins associated with the IM and the extracellular space were exclusively identified in Δ*tolA OMVs* and at two to three-fold higher quantities than in WT OMVs (Figure 4B). This strongly supports the presence of greater IM, periplasmic, and extracellular protein carryover in Δ*tolA* OMVs, in agreement with our cryo-TEM multi-lamellar vesicle images. Additionally, many of the overlapping proteins identified in both WT and Δ*tolA* vesicle proteomes were located in the OM, including porins (OmpA, OmpC, and OmpF), lipoproteins (LolB, Lpp, RcsF, RlpA, and SlyB), and membrane assembly proteins (BamB and LpoA) (Figure 4C and Table 1). Membrane integrity proteins (Pal and TolB) as well as stress-related proteins (Dps, HdeB, and OsmY) were noted in both WT and Δ*tolA* OMV proteomes (Table 1), indicating that membrane components involved in stress and membrane maintenance were present even in the WT vesicles. Some of the proteins we detected were previously identified in other OMV proteomic studies (Lee et al., 2007; Berlanda Scorza et al., 2008; Aguilera et al., 2014; Kim et al., 2018; Hong et al., 2019), as well as others implicated in studies pertaining to OMV formation (McBroom et al., 2006; McBroom and Kuehn, 2007; Schwechheimer et al., 2015). Such proteins included periplasmic chaperone/protease DegP and the OM-anchored lipoprotein NlpE, which were found exclusively in the Δ*tolA* OMV sample (Table 1).

Functional analysis of OMV proteomes from WT and Δ*tolA* strains was performed using the Kyoto Encyclopedia of Genes and Genomes (KEGG) database (Supplementary Figure 2). This analysis revealed enrichment of a number of proteins associated with two-component regulatory systems, flagellar proteins, ribosomal proteins as well as several metabolic pathways, predominantly from the Δ*tolA* OMV sample (Supplementary Figure 2). Together, this proteomic analysis shows that both WT and Δ*tolA* strains produce vesicles enriched with proteins that are highly membrane-associated, that are responsible for membrane trafficking and assembly, and play a role in membrane integrity and bacterial stress response. It also verifies that deletion of *tolA* results in OMV formation with much greater periplasmic, IM and extracellular content carryover than WT OMVs as suggested by cryo-TEM imaging.

## Discussion

This study determined that both UC and UF OMV isolation methods were effective for isolating intact OMVs, as both methods yielded comparable WT and Δ*tolA* vesicle populations (Figure 3). It did reveal that UC and UF vesicles differed with respect to their specific OMV size ranges and size distributions (Figures 1B, 3). It also revealed some differences in total protein and phosphorous content quantifications of WT and Δ*tolA* OMVs (Figure 2). These findings are important when considering how any isolated OMVs will be experimentally studied. OMVs isolation is a time-consuming process that requires large volumes of culture to overcome lower yields of vesicles naturally produced by cultured bacteria. While several authors have sought to optimize current methods to achieve the highest yields of vesicles (Cvjetkovic et al., 2014; Klimentová and Stulík, 2015; van der Pol et al., 2015), to our knowledge this is the first study to directly compare two of the most widely used isolation methods for their experimental OMV analysis applications. Despite both methods isolating high purity OMVs, our findings show that UF improves the recovery of OMVs as compared to UC isolations, without qualitatively altering vesicle contents or morphology. In addition, the UC approach is more time-consuming as compared to UF due to the duration of centrifugation runs and was shown in this study to reduce the recoverable quantity of smaller sized vesicles (Supplementary Figure 1). However, it is important to note that UC did enhance isolation of larger sized vesicles (>100 nm) (Supplementary Figure 1). As a result, our study offers more insights into the benefits and limitations of each technique, which should be considered in future OMV isolation experiments. It is important to note that many OMV isolation methods involving either UC or UF must be performed under sterile conditions to avoid foreign particle contamination which may obscure downstream analyses. Additionally, many OMV isolation studies include an extra OMV purification step such as gel filtration or gradient ultracentrifugation to further purify and enrich for vesicles of defined sizes or molecular weights as reviewed by Klimentová and Stulík (2015). We chose to omit gradient ultracentrifugation and gel filtration method assessments herein, as these methods require some prior knowledge of vesicle size distributions and would have prevented our unbiased assessment of vesicle size ranges obtainable by these initial UC or UF OMV isolation techniques.

### Smaller Sized Vesicles Are Enriched by UF

The average vesicle size and size distribution as determined by NTA was generally in agreement with the cryo-TEM analysis data for both UC and UF OMVs, but we did notice differences in the size of particles isolated by each method. Based on NTA results, UF-isolated vesicles from WT and Δ*tolA* had a higher proportion of smaller (<100 nm) vesicles, while UC-isolated samples from WT and Δ*tolA* had a higher proportion of vesicles of larger (>100 nm) sizes (Supplementary Figure 1A). This trend in size distribution was also observed when we compared the Δ*tolA* vesicles in cryo-TEM measurements, where UF samples had a smaller average size, however, no significant differences were found between WT UC and UF sizes according to cryo-TEM images (Supplementary Figure 1B). This result suggests that NTA may be detecting small fragments of particles rather than fully formed OMVs in the UF-isolated samples. In a previous study it was found that NTA can measure vesicles as small as ∼50 nm (Dragovic et al., 2011), however, we noticed that smaller particles were being measured by the NTA NanoSight instrument in both UC and UF samples. Therefore, the smaller particles (0–50 nm in diameter) could be undissolved salts/media components and/or cell debris particles that were carried over from cell cultures in UF isolations, potentially skewing the size distribution of vesicles in samples with smaller contaminating particles. We suspect that the UF procedure may naturally bias isolation of smaller sized particles as the medium is membrane filtered, and may be prone to some filter blockage by medium components and culture carryover over time. Filter blockage may cause the MW cut-off to become lower over time and allow smaller sized particles, such as salt or cell debris, to build up and enrich in UF techniques. Sucrose-gradient cushions have been used in past experiments to help eliminate these carryover particles (Alexander et al., 2016) but it is unclear if cushions further bias the recovery of heterogenous OMV populations.

Another explanation for why UF isolates smaller OMVs than UC methods is that UC methods may promote aggregation of vesicles due to repeated pelleting steps. Vesicle pelleting may result in larger average sized OMV distributions as we observed in our study. Aggregation of vesicles and contamination with extravesicular protein complexes or aggregates is a commonly reported UC occurrence (for examples see Théry et al., 2006; Issman et al., 2013; Witwer et al., 2013; Linares et al., 2015). Due to the use of fixed-angle ultracentrifuge rotors during UC, material pelleting and deposition against the wall of the centrifuge tube may be physically damaging, potentially promoting vesicle aggregation, which favors the fusion of vesicles with weakened or altered membranes (Witwer et al., 2013; Klimentová and Stulík, 2015). In our study, we detected a small fraction of UC vesicles larger than 450 nm by NTA, which suggests this form of aggregation was occurring, as the 0.45 μm filtration step prior to UC/UF should remove all particles above this size. Thus, while UC and UF methods are both comparable, it appears that each method alters the size distribution of OMVs, with UF selecting for smaller particles and UC promoting the aggregation of vesicles into larger-sized particles.

### Δ*tolA* OMVs Are Multilamellar and Enriched With IM-Associated Proteins

Mutations of the *tol-pal* system genes have been well documented in OMV studies and mutants are often used study hypervesiculation phenotypes (Bernadac et al., 1998; Kulp et al., 2015), however, our study is the first to examine the morphology and content of a *tolA* mutant in detail. Our study determined that the Δ*tolA* strain not only produced more OMVs as compared to the WT strain, but that these vesicles were larger in size and displayed M-OMV, G-OMV, and O-IMV membrane morphology as observed for *tolB* mutant of *B. agrestis* in a recent study (Takaki et al., 2020). Additionally, we determined that Δ*tolA* vesicles with multiple membranes were larger in size than WT OMVs. The presence of these unique O-IMV and multi-lamellar vesicle types (M-OMV and G-OMV) has also been observed for many Gram-negative species such as *E. coli* Nissle 1917, *Helicobacter pylori* strain 60190, *Pseudomonas aeruginosa* PAO1, *Acinetobacter baumannii* AB41, and *Neisseria gonorrhoeae* DSM15130 strains, but their functional significance to these species has yet to be determined (Fiocca et al., 1999; Pérez-Cruz et al., 2015, 2016). This altered Δ*tolA* vesicle morphology suggests that the loss of TolA in *E. coli* promotes the carryover of IM, possibly to compensate for loss of membrane integrity caused by reduced Tol-Pal inter-membrane connections. Tol-Pal mutants have been previously shown to cause cell division impairments, resulting in increased distance between the IM and the peptidoglycan layer and enhancing defects in peptidoglycan-cleaving enzymes (Takaki et al., 2020; Yakhnina and Bernhardt, 2020). Our proteomic data reveals that the peptidoglycan degrading enzymes NlpD, MltA, Slt, and RlpA were significantly enriched in the Δ*tolA* strain (Table 1), supporting the idea that the Tol-Pal system plays a role in promoting glycan cleavage. Similar to the recent Δ*tolB* study (Takaki et al., 2020), we suspect that increased OMV formation by the Δ*tolA* mutant is the result of incomplete tethering of the IM and OM, which would result in IM and cytoplasmic proteins being carried over into vesicles more frequently. This is supported by our proteomic analysis, which determined that more cytoplasmic proteins were detected in Δ*tolA* than WT, and IM proteins were found exclusively in the Δ*tolA* vesicles (Figure 4B). It is important to note that the detection of cytoplasmic proteins in both the WT and Δ*tolA* OMVs is not unexpected, since cytoplasmic protein detection in OMV isolations has been previously shown to occur in many past OMV studies, as reviewed by Nagakubo et al. (2020). There are two explanations for this, the first is that there are typically a small fraction of vesicles produced by a bacterium (∼0.1%) that contain both inner and outer membranes as well as carry over cytosolic proteins that remain detectable by proteomic analyses (Pérez-Cruz et al., 2013). Since many cytoplasmic proteins are present in the cell at higher amounts than many membrane proteins, cytoplasmic protein detection in OMV preparations is not surprising. Secondly, *Pseudomonas* OMV studies also suggest that cytosolic protein carryover may be the result of transient autolysin-induced peptidoglycan breakage which not only promotes some inner membrane carry over into vesicles but also likely carries over cytoplasmic proteins (Kadurugamuwa and Beveridge, 1995; Clarke, 2018). Hence, the detection of some cytoplasmic proteins even in the WT OMV samples of our study was expected.

Our isolated Δ*tolA* vesicles also appeared to be enriched with structural OMPs, cell membrane assembly proteins, and cell division proteins. Our proteomic analysis determined that Δ*tolA* OMVs were enriched with proteins involved in membrane biogenesis and degradation, including the OM assembly proteins (BamC and BamD), LPS assembly proteins (LptD, LptE, and FadL), cell division proteins (CpoB, DamK, and NlpD), and murein degrading proteins (MltA, MipA, and Slt) (Figure 4C and Table 1). When compared to the WT, this suggests that the Δ*tolA* mutant may have to increase membrane biogenesis as well as membrane turnover in order to keep up with the high levels of vesiculation and loss of both IM and OM to vesicles caused by Tol-Pal complex disruption. Our measurements of total phosphate and protein in the Δ*tolA* strain by both isolation methods indicated that more protein was detected relative to total lipid phosphate, which further supports this explanation. Additionally, many envelope stabilizing proteins were also enriched in Δ*tolA*, specifically NlpE, OmpX, and TolC, which support increased membrane biogenesis in *tolA* mutant vesicles (Figure 4C and Table 1). The increased prevalence of these proteins in Δ*tolA* OMVs is not surprising, as previous studies have shown that mutations in *tolA* can be partially compensated by expressing other OM-associated proteins that act to stabilize the OM in the absence of TolA (Godlewska et al., 2009; Bager et al., 2013; Pérez-Cruz et al., 2016). An increase in the amount of stabilizing proteins in the OM could conceivably compensate for the increased vesiculation in mutant strains and is worth further study. We also observed that the Δ*tolA* strain grows slower than the WT (Figure 1A), which underscores the fitness costs associated with loss of TolA and inter-membrane integrity in *E. coli*.

### The Role of the Tol-Pal System in Vesicle Production

The modulation of cell envelope intermembrane layer crosslinks is a strong correlate of increased OMV production in Gram-negative species. Proteins intricately involved in linking the OM to the IM include: (i) OmpA, an OM porin that spans the periplasmic space and can bind to peptidoglycan. (ii) The Tol-Pal complex, a cell-division component that aids in invagination of the OM and membrane stability. (iii) Lpp, an OM lipoprotein that covalently crosslinks with the peptidoglycan (Schwechheimer and Kuehn, 2015). Studies have shown that mutants of *E. coli, Salmonella*, and *A. baumannii* lacking OmpA display increased OMV production (Deatherage et al., 2009; Schwechheimer and Kuehn, 2015). Mutations in the Tol-Pal genes are associated with increased vesicle production in *E. coli* and *Salmonella*, specifically deletions in *pal, tolA*, and *tolB* (Cascales et al., 2002; Deatherage et al., 2009; Jagannadham and Chattopadhyay, 2015). Our study corroborates these findings, with OmpA, Lpp, TolB, and Pal all enriched in the Δ*tolA* mutant (Table 1), suggesting that the hypervesiculation phenotype exhibited by our Δ*tolA* strain is a result of generalized membrane instability and incomplete membrane linkage.

Proteomic analysis of Δ*tolA* OMVs also identified the involvement of σ^*E*^ and Cpx envelope stress response pathways (Figure 4C and Table 1). In the presence of misfolded proteins and extracellular stress, the σ^*E*^ response is activated, and contributes to DNA repair, metabolism, OM biogenesis, and periplasmic homeostasis (Hews et al., 2019). σ^*E*^-regulated chaperones and proteases (SurA, FkpA, and DegP) and several members of the BAM complex (BamB, BamC, and BamD) were all significantly enriched in Δ*tolA*, indicating that this pathway is highly active in the Δ*tolA* mutant. The chaperone Skp was also found in both WT and Δ*tolA*, although we did not significantly detect over-accumulation in either sample. The Cpx envelope stress response is crucial for mitigating envelope stress caused by misfolded proteins in the periplasm, and Cpx regulated members are involved in protein folding and degradation primarily within the IM (Danese et al., 1995; Snyder et al., 1995; Danese and Silhavy, 1998). NlpE, an activator of this system, was found to be significantly enriched in Δ*tolA* only (Figure 4C and Table 1). DegP, a periplasmic serine protease was the only significantly enriched protein regulated by the Cpx regulon. It is important to note, that the Cpx regulon has been associated with IM-associated proteins and functions (Raivio et al., 2013), as was seen in our Δ*tolA* proteomic dataset. Together, this suggests that mutations in the Tol-Pal system are intricately involved with envelope stress responses as a compensatory mechanism against envelope instability.

## Conclusion

The study of OMVs is a rapidly expanding research area, so understanding the isolation method limitations improves our ability to modulate OMV production using the fewest genetic alterations. Better understanding of OMV recovery by common vesicle isolation methods aids ongoing and future biotechnological OMV applications, helping to standardize and improve efforts to enhance the overall recovery of OMVs from bacterial cultures. Our analyses suggest that UF may be an improved method for isolating OMVs, due to its faster isolation time and higher yield of smaller and averaged-sized vesicles. Depending on the OMV sizes desired, UC applications may be a desired methodology and both methods should be carefully considered based on the type of downstream experimental analysis needed. Our study also provides the first in-depth characterization of Δ*tolA* OMVs, which revealed a multi-lamellar membrane morphology similar to recent studies of *tolB* (Takaki et al., 2020). Our proteomic analysis highlighted the impact that the Tol-Pal system has on cell membrane content released into secreted vesicles and identified protein components worth following up on in future studies.

## Materials and Methods

### Bacterial Strains

The parental Keio collection strain *E. coli* K-12 BW25113 (WT) and its single gene deletion mutant JW0729 (Δ*tolA*) were obtained from the Coli Genetic Stock Center (Yale University, New Haven, CT, United States). All strains were grown in Luria-Bertani (LB) broth (Cold Spring Harbor Protocols, 2006) in a shaking incubator 170 RPM at 37°C from overnight cultures of cryopreserved dimethylsulfoxide (DMSO) stocks. Growth was monitored by measuring optical density (OD) at 600 nm (OD_600 *nm*_). All procedures involved the use of a biosafety cabinet to maintain sterility of bacterial and OMV isolates and prevent their contamination.

### Growth Rate Measurements

To ensure that OMV production was not associated with impaired growth phenotypes, growth curves of the WT and Δ*tolA* strain were performed. Bacterial cells were inoculated into LB broth from frozen cryostocks and grown overnight. The resulting culture was standardized to 1 OD, and diluted 1/100 in LB into flat-bottom 96-well NUNC microtiter plates (Thermo Fisher Scientific, United States). Strains were grown for 24 h (h) in LB media at 37°C with continuous shaking, where OD_600 *nm*_ was measured every 2 h in a BioTek EL808 microplate reader (BioTek, Winooski, VT, United States). Growth of each strain was measured in triplicate from 6 biological replicates (*n* = 6) and Mann–Whitney *U* tests were performed to determine OD values that differed significantly (*p* < 0.05) between WT and Δ*tolA* at all time points.

### OMV Isolation

#### Culture Supernatant Separation Prior to UC and UF

Prior to UC or UF OMV isolation, both methods separated supernatants prepared from large scale LB cultures as described previously (Horstman and Kuehn, 2000; McBroom et al., 2006; Lee et al., 2007) with slight modifications. Briefly, bacterial cells were inoculated into LB broth from frozen cryostocks and grown overnight. The resulting culture was standardized to 1 OD_600 *nm*_ unit, washed two times to prevent carryover of OMVs, and diluted 1/100 into 1 L of LB broth. This culture was incubated at 37°C for 18 h with constant shaking (160 RPM). OD_600 *nm*_ measurements were taken to confirm early stationary growth phase OD_600 *nm*_ values in reference to the growth curves performed in Section “Growth Rate Measurements.” Cells were separated from the culture by centrifugation at 6,000 RPM for 15 min at 4°C in an JLA9.1000 rotor using an Avanti J-E high speed centrifuge (VWR Part of Avantor, United States). The collected supernatant was filtered with a 0.45 μm polyethersulfone (PES) vacuum filter (MilliporeSigma, United States) to remove any residual bacteria. Filtered supernatant aliquots from each strain preparation were spread plated onto LB agar and incubated at 37°C for 24 h to confirm the absence of intact, viable cells. The resulting filtrate was divided into two equal parts to be assessed by both ultracentrifugation (UC) and ultradiafiltration (UF) methods.

#### Ultracentrifugation (UC) OMV Isolation

The designated UC filtered supernatant portion was centrifuged at 40,000 RPM in polycarbonate tubes for 2 h at 4°C in a Ti70 rotor using a Beckman Coulter^®^ Optima XPN Ultracentrifuge (VWR Part of Avantor, United States). The supernatant was carefully decanted to prevent pellet disruption. After all the filtrate had been centrifuged, the pellets in each tube were resuspended in 50 mM HEPES buffer (Fisher Scientific, NH, United States) then stored at −20°C (Yaron et al., 2000).

#### Ultradiafiltration (UF) OMV Isolation

The designated UF filtered supernatant portion was concentrated 50-fold in 50 mM HEPES buffer in a 400 ml capacity Amicon^®^ stirred cell (MilliporeSigma, United States) ultradiafiltration system using a 500 kiloDalton (kDa) molecular weight cut off (MWCO) polyethersulfone (PES) ultrafiltration disk (MilliporeSigma, United States). The concentrated retentate was collected and divided into polycarbonate centrifuge tubes (Beckman) before ultracentrifugation at 40,000 RPM for 2 h at 4°C. The pellet was resuspended in 50 mM HEPES buffer and stored at −20°C until further use (Yaron et al., 2000).

### Protein and Lipid Quantification of OMVs

Outer membrane vesicles produced by WT and Δ*tolA* bacteria were assessed using both protein and phosphate assays to quantify the amount of OMVs produced by each strain and each method. Protein concentration was measured by a bicinchoninic acid (BCA) assay (Thermo Fisher Scientific, United States). Samples were measured in triplicate and compared with a standard curve plotted using serial dilutions of bovine serum albumin (BSA). Lipid content was inferred by measuring the total phosphate content using a malachite green phosphate assay (MilliporeSigma, United States). This assay quantifies the amount of phosphate in phospholipids, protein, and DNA. Assays were performed in triplicate for each OMV isolation. Protein- or phosphate-based measurements were adjusted for the amount of bacteria in the culture and for the vesicle production of the wild-type culture according to the following two equations.

EQ1a: [Protein]_*Sample*_ = (OD_562 *nm*_ − *B*)/*A*

EQ1b: [PO_4_^3–^]_*Sample*_ = (OD_620 *nm*_ − *B*)/*A*

Equation 1 (EQ1) calculates the OMV sample’s protein and phosphate concentrations based on standard curves of bovine serum albumin (BSA) protein (EQ1a) and potassium phosphate (EQ1b) titrations, where the sample OD unit value at the measured wavelength (562 nm or 620 nm) is subtracted from the respective absorbance y-intercept (B) value from the standard curve, which is divided by the slope of the respective standard curve (A).

EQ2a: Adjusted [Protein]_*Sample*_ = [Protein]_*Sample*_/original culture CFU/mL

EQ2b: Adjusted [PO_4_^3–^]_*Sample*_ = [PO_4_^3–^]_*Sample*_/original culture CFU/mL

Equation 2 (EQ2) adjusts for the number of cells in the original culture by dividing the OMV sample’s protein or phosphate concentrations determined from EQ1 by the OD _600 *nm*_ of the original culture converted to colony forming units (CFU) per mL that the OMV isolations were obtained from.

### Tricine Sodium Dodecyl Sulfate-Polyacrylamide Gel Electrophoresis (Tricine SDS-PAGE) and Western Blot Analysis of OMV Proteins

To determine whether the protein content of OMVs isolated by each method may differ due to the isolation methods themselves, Tricine sodium dodecyl sulfate-polyacrylamide gel electrophoresis (Tricine SDS-PAGE) was used. OMV preparations were denatured for 10 min at 65°C in 2 × Laemmli buffer [100 mM dithiothreitol, 150 mM Tris (pH 7), 12% w/v SDS, 30% w/v glycerol, 0.05% w/v Coomassie Brilliant Blue G-250] and equal amounts with respect to total protein quantities from both preparations were separated by 12% Tricine SDS-PAGE gels. Proteins were visualized with 0.5% v/v 2,2,2-trichloroethanol (TCE) by ultraviolet detection (Ladner et al., 2004). For Western blot, proteins were transferred to nitrocellulose and then blocked with 5% milk powder in TBS for 1 h. The membrane was incubated with anti-Gram-negative bacterial OmpA primary antibody at 1:25000 dilution (1.2 μg/mL; Antibody Research Corporation, Cottleville, MO, United States) overnight at 4°C followed by incubation with goat anti-rabbit IgG (Heavy + Light) HRP conjugate antibody at a 1:500 dilution (0.4 μ/mL; Life Technologies, United States) for 1 h. Proteins were detected using an enhanced chemiluminescence (ECL) detection system kit (Thermo Fisher Scientific, United States). Blot band densities were densitometrically analyzed using ImageJ software version 1.51^1^ and quantified as described in Davarinejad (2020).

### Nanoparticle Tracking Analysis (NTA) of OMVs

Outer membrane vesicles quantities and sizes were determined using a NanoSight NS500 nanoparticle tracking (NTA) instrument (Malvern Instruments Ltd., United Kingdom) equipped with a 488 nm blue laser and a complementary metal-oxide semiconductor (CMOS) image sensor camera. OMV samples were thawed to room temperature prior to analysis and diluted 1/1000 in 50 mM HEPES buffer at pH 7.4. Polystyrene beads (100 nm diameter) and HEPES buffer alone were run as positive and negative control standards, respectively. Samples were infused into the NanoSight instrument using a syringe pump set at ‘20’ speed setting (in arbitrary units). Measurements were captured in five 60 s reads at ambient room temperature (23.9–25.2°C), with instrument-optimized settings, where ‘blur,’ ‘minimum track length,’ and ‘minimum expected size’ options were set to “automatic” and viscosity was set to “water” (0.883–0.911 cP). Automated image setup (camera level and focus) was chosen whenever available for video enhancement. A total of 1,498 frames per sample were analyzed with NTA software version 2.3 (Malvern Instruments Ltd., United Kingdom) with a detection threshold of 5 (in arbitrary units). Mean size (nm), mode size (nm), and concentration (particles/mL) were tabulated, and the average of five reads was calculated and plotted as particle size versus number of particles per mL.

### Cryo-TEM Analysis of Isolated OMVs

Samples for cryo-TEM were prepared as described above for OMV isolation, with the exception that UC and UF Δ*tolA* OMV samples were diluted 10-fold in 50 mM HEPES buffer (pH 7.4) due to their higher concentration of OMVs when compared to WT samples. All sets of samples were combined with 10 nm BSA-labeled gold tracer in a 6:1 ratio to assist with automated focusing; 3 μL of this suspension was applied to freshly glow-discharged Quantifoil R 2/2 grids (Quantifoil Micro Tools GmbH, Germany). This suspension was allowed to adhere, and the excess liquid was blotted with standard Vitrobot filter paper (Ted Pella Inc., United States) using a Vitrobot Mark IV (Thermo Fisher Scientific, United States), operating at 5°C and 100% humidity. Grids were then frozen in liquid ethane cooled by liquid nitrogen. Samples were transferred to a Tecnai F20 transmission electron microscope (Thermo Fischer Scientific, United States) using a Gatan 626 DH low-temperature specimen holder (Gatan Inc., United States), and images were recorded using an Eagle 4k CCD camera (Thermo Fischer Scientific, United States). Images were taken in low-dose imaging conditions (10 e/Å^2^) at both 5,000 and 14,500× magnifications, and vesicle sizes and morphologies were analyzed using ImageJ software version 1.51^2^.

### Proteomic Analysis and Gene Ontology

#### Sample Preparation and Nano LC-MS/MS

Samples for proteomic analysis were prepared from 1 L cultures as described for OMV isolation using the UF method for concentration. Protein from the outer membrane vesicles (OMV) were quantified using a bicinchoninic acid (BCA) protein assay kit, with bovine serum albumin (BSA) as the standard (Thermo Fisher Scientific, United States). SDS was added to 100 μg of OMV protein at a final concentration of 2%, and then heated at 95°C for 5 min. Upon cooling to room temperature, dithiothreitol was added to a final concentration of 100 mM, and heated at 95°C for 5 min. Samples were frozen at −80°C until ready for use. A total of 100 μg of protein from each OMV sample was used for each digestion. Protein samples were digested with trypsin (Promega, United States) overnight using a filter-assisted sample preparation (FASP) method described previously (Wiśniewski et al., 2009) using Nanosep 30K Omega Centrifugal Devices (Pall Corporation, United States). Following digestion, all samples were dried down and reconstituted using mass spectrometry grade water to a final concentration of 0.5μg/μl prior to mass spectrometry analysis.

Samples were each separately analyzed using a nano-flow Easy nLC II connected in-line to an LTQ Orbitrap Velos mass spectrometer with a nanoelectrospray ion source at 2.2 (Thermo Fisher Scientific, United States). Peptide samples (2 μl) were loaded onto a C18-reversed phase trap column (3 cm long, 100 μm inner diameter, 5 μm particles) with 100% buffer A (2% acetonitrile, 0.1% formic acid) for a total volume of 30 μl, and then separated on a C18-reversed phase column (15 cm long, 75 μm inner diameter, 2.4 μm particles). Both columns were packed in-house with ReproSil-Pur C18-AQ resin (Dr. Maisch) and fritted with Kasil. Peptides were eluted using a linear gradient of 5–25% buffer B (98% acetonitrile, 0.1% formic acid) over 120 min, 25–40% buffer B for 5 min, 40–80% buffer B for 5 min and a wash at 80% B for 8 min at a constant flow rate of 250 nl/min. Total LC/MS/MS run-time was about 165 min, including the loading, linear gradient, column wash, and the equilibration.

Data was acquired using these settings: dynamically choosing the top 10 most abundant precursor ions from each survey scan, each isolated with a width 2.0 m/z and fragmentation by CID. The survey scans were acquired in the Orbitrap over m/z 300–1,700 with a target resolution of 60,000 at m/z 400, and the subsequent fragment ion scans were also acquired in the iontrap at a normal scan rate. The lower threshold for selecting a precursor ion for fragmentation was 2000. Dynamic exclusion was enabled using a m/z tolerance of 15 ppm, a repeat count of 1, and an exclusion duration of 30 s.

#### Data Processing

All spectra were processed using MaxQuant (v1.6.7, Max Plank Institute) using the imbedded Andromeda search engine. Searches were performed against a subset of the SwissProt database set to *E. coli* K12 (4519 sequences). The following search parameters were used: Carbamidomethyl (C) was selected as a fixed modification, Oxidation (M) and Acetyl (Protein N-term) as a variable modifications, fragment ion mass tolerance of 0.5 Da, parent ion tolerance of 20 ppm, and trypsin enzyme with up to 2 missed cleavage. False discovery rates were set up using 0.01 for peptides, 0.01 for proteins, and at least 1 razor peptide per protein. LFQ was enabled for Quantitation. Resulting LFQ intensities were imported into Perseus v1.6.5 (Max Plank Institute). In Perseus the data was Log2 transformed. Then all the proteins that did not have a least 3 valid log2 LFQ intensities from ID were filtered out.

All spectra were also processed using Proteome Discoverer (v2.2, Thermo Fisher Scientific) and database searching was done with Mascot v2.6 (Matrix Science). Searches were performed against the SwissProt database (2020_01) (5461,911 sequences) The decoy database option was selected, and the following search parameters were used: Carbamidomethyl (C) was selected as a fixed modification, Oxidation (M) as a variable modification, fragment ion mass tolerance of 0.5 Da, parent ion tolerance of 10 ppm, and trypsin enzyme with up to 1 missed cleavage. Mascot search results were imported into Scaffold Q + (v4.11.0). Proteins were filtered using a 1.0% false discovery rate and assessed for significance using Fisher’s exact test (*p*-value < 0.05). All significant proteins were annotated by their subcellular localization using the pSORTb algorithm (Yu et al., 2010). String protein networks were constructed using Cytoscape (version 3.8.0^3^), and functional protein maps were constructed using the Kyoto Encyclopedia of Genes and Genomes (KEGG) database through the ClueGo plugin (v2.5.6^4^) with default settings. The mass spectrometry proteomics data have been deposited to the ProteomeXchange Consortium via the PRIDE (Perez-Riverol et al., 2019) partner repository with the dataset identifier PXD022786 and 10.6019/PXD022786.

### Statistical Analysis

All data was analyzed using Graph Pad Prism 8 software (v8.4.2). Normality of data was assessed for cryo-TEM and NTA data using the Shapiro–Wilk and Kolmogorov–Smirnov test. Statistical significance for all data was determined by the Mann–Whitney *U* test. For all analyses, differences between either WT and Δ*tolA* preparations or between the same strain isolated by UC or UF were statistically compared, and results with a *p*-value of less than 0.05 were considered statistically significant due to sample numbers compared and their degrees of freedom.

## Data Availability Statement

The mass spectrometry proteomic data presented in the study are deposited in the ProteomeXchange Consortium via the PRIDE partner repository with the dataset identifier PXD022786 and 10.6019/PXD022786.

## Author Contributions

DCB and SR designed the study. SR isolated the OMVs and performed growth curves, protein/phosphate assays, and western blots. SR completed the cryo-TEM imaging and NanoSight experiments with DRB, SH, and TB. SR prepared proteomic sample preparations for LC MS/MS collection by PC and GW. SR analyzed all data and prepared manuscript figures. SR wrote the manuscript drafts in consultation with DCB, where DCB and GGZ edited. All authors read and approved the final manuscript.

## Conflict of Interest

The authors declare that the research was conducted in the absence of any commercial or financial relationships that could be construed as a potential conflict of interest.

## Footnotes

1 https://imagej.nih.gov/ij/

2 https://imagej.nih.gov/ij

3 https://cytoscape.org

4 http://apps.cytoscape.org/apps/cluego

## Supplementary Material

Supplementary Figure 1 Size of isolated OMVs by NanoSight NTA and cryo-TEM. OMVs isolated from WT and Δ*tolA* were binned in 50 nm size ranges and represented as a percentage of the total OMV population for **(A)** NTA, **(B)** Cryo-TEM.

Supplementary Figure 2 KEGG network of WT and Δ*tolA* OMV-associated proteins. Proteins were functionally annotated using the ClueGO (v.2.5.6) add-in for Cytoscape (v.3.8.0) (Bindea et al., 2009). Bolded black terms with black nodes represent functional categories, and colored nodes represent proteins with the color of each node indicating the OMV sample it was found in. Proteins were exclusive to WT (blue), Δ*tolA* (green) or present in both samples (purple). Connecting edge lines represent multiple functional categorizations of proteins. Default parameters were used for network specificity.
