# Supplementary material for: Comparative Analysis of Outer Membrane Vesicle Isolation Methods With an Escherichia coli tolA Mutant Reveals a Hypervesiculating Phenotype With Outer-Inner Membrane Vesicle Content
Source: Front Microbiol. 2021 Mar 5;12:628801. doi: 10.3389/fmicb.2021.628801 (PMC7973035; doi:10.3389/fmicb.2021.628801)
Supplement: Supplementary Figure 1 — Size of isolated OMVs by NanoSight NTA and cryo-TEM. OMVs isolated from WT and ΔtolA were binned in 50 nm size ranges and represented as a percentage of the total OMV population for (A) NTA, (B) Cryo-TEM. [file Data_Sheet_1.PDF]

Supplementary files for the article titled:

Comparative analysis of outer membrane vesicle isolation methods with an *Escherichia coli tolA* mutant reveals a hypervesiculating phenotype with outer-inner membrane vesicle content

By authors: Shelby L. Reimer[1], Daniel R. Beniac[2], Shannon L. Hiebert[2], Timothy F. Booth[2], Patrick M. Chong[2], Garrett R. Westmacott[2], George G. Zhanel[1], Denice C. Bay[1§]

[1.] Department of Medical Microbiology and Infectious Diseases, University of Manitoba, Winnipeg, Manitoba, Canada

[2.] National Microbiology Laboratory, Public Health Agency of Canada, Winnipeg, Manitoba, Canada

[§]Corresponding author:

Denice C. Bay
Assistant Professor
Rm 514C Basic Medical Sciences Bldg.
Department of Medical Microbiology and Infectious Diseases
University of Manitoba
745 Bannatyne Avenue
Winnipeg, MB, Canada R3E 0J9
Tel: (204) 977-5679
Fax: (204) 789-3926
Email: Denice.Bay@umanitoba.ca

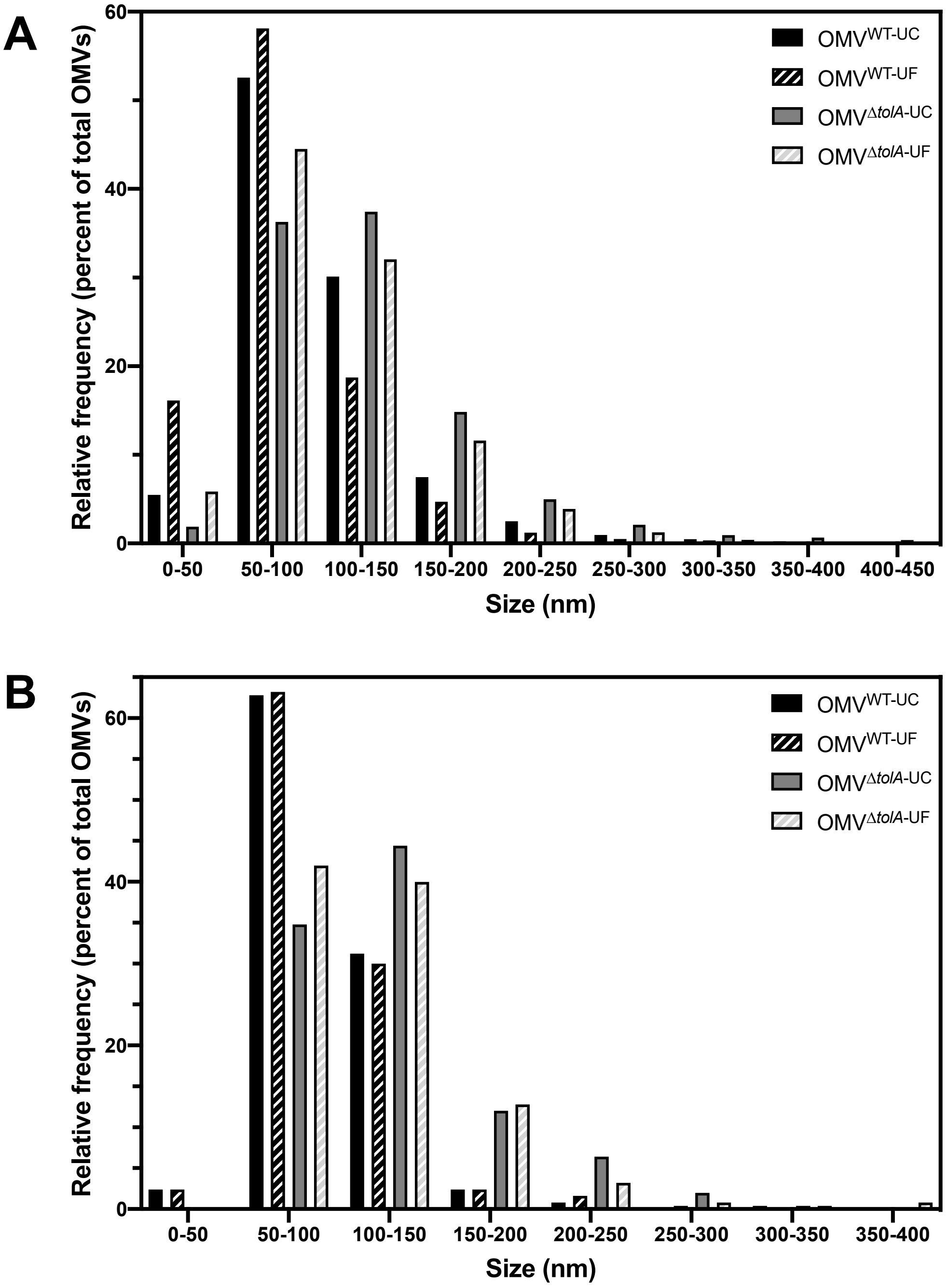

Supplementary Figure S1. Size of isolated OMVs by NanoSight NTA and cryo-TEM. OMVs isolated from WT and Δ*tolA* were binned in 50 nm size ranges and are represented as a percentage of the total OMV population measured by A) NTA, and B) Cryo-TEM.

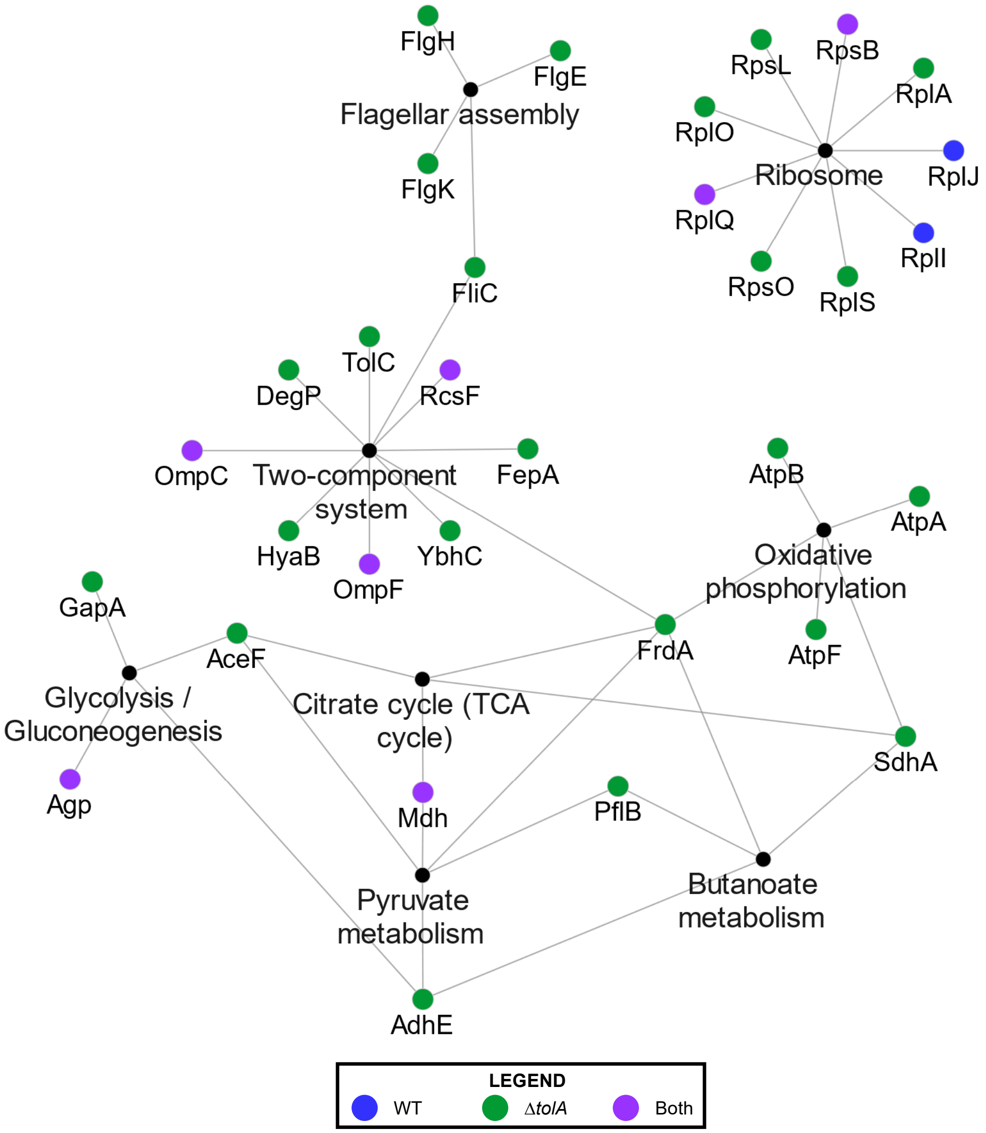

Supplementary Figure S2. KEGG network of WT and Δ*tolA* OMV-associated proteins. Proteins were functionally annotated using the ClueGO (v.2.5.6) add-in software for Cytoscape (v.3.8.0) (1). Bolded black terms below black nodes represent functional categories, and coloured nodes represent proteins with the colour of each node indicating the OMV sample it was found in. Proteins were exclusive to WT (blue), Δ*tolA* (green) or present in both samples (purple). Connecting edge lines represent multiple functional categorizations of proteins. Default parameters were used for network specificity.

Reference:

1. Bindea G, Mlecnik B, Hackl H, Charoentong P, Tosolini M, Kirilovsky A, Fridman WH, Pagès F, Trajanoski Z, Galon J. 2009. ClueGO: A Cytoscape plug-in to decipher functionally grouped gene ontology and pathway annotation networks. Bioinformatics 25:1091-3.
